# Glioma surveillance imaging: current strategies, shortcomings, challenges and outlook

**DOI:** 10.1259/bjro.20200009

**Published:** 2020-06-23

**Authors:** Gehad Abdalla, Ahmed Hammam, Mustafa Anjari, Dr. Felice D'Arco, Dr. Sotirios Bisdas

**Affiliations:** 1 Department of Neuroradiology, The National Hospital for Neurology and Neurosurgery, University College London Hospitals NHS Trust, London, UK; 2 Department of Neuroradiology, Great Ormond Street Hospital for Children, London, UK; 3 Department of Brain Repair and Rehabilitation, Institute of Neurology, University College London, London, UK

## Abstract

Inaccurate assessment of surveillance imaging to assess response to glioma therapy may have life-changing consequences. Varied management plans including chemotherapy, radiotherapy or immunotherapy may all contribute to heterogeneous post-treatment appearances and the overlap between the morphological features of pseudoprogression, pseudoresponse and radiation necrosis can make their discrimination very challenging. Therefore, there has been a drive to develop objective strategies for post-treatment assessment of brain gliomas. This review discusses the most important of these approaches such as the RANO “Response Assessment in Neuro-Oncology”, iRANO “Immunotherapy Response Assessment in Neuro-Oncology” and RAPNO “Response Assessment in Paediatric Neuro-Oncology” models.

In addition to these systematic approaches for glioma surveillance, the relatively limited information provided by conventional imaging modalities alone has motivated the development of novel advanced magnetic resonance (MR) and metabolic imaging methods for further discrimination between viable tumour and treatment induced changes. Multiple clinical trials and meta-analyses have investigated the diagnostic performance of these novel techniques in the follow up of brain gliomas, including both single modality descriptive studies and comparative imaging assessment. In this manuscript, we review the literature and discuss the promises and pitfalls of frequently studied modalities in glioma surveillance imaging, including MR perfusion, MR diffusion and MR spectroscopy. In addition, we evaluate other promising MR techniques such as chemical exchange saturation transfer as well as fludeoxyglucose and non-FDG positron emission tomography techniques.

## Introduction

Neuroradiological assessment of brain gliomas plays a crucial role in their primary diagnosis andtheir post-therapeutic follow-up. In low-grade gliomas (LGG), imaging is pivotal in monitoring tumour stability and evaluation for possible anaplastic transformation. Surgical excision of gliomas is not always an option in tumours in highly eloquent areas. Consequently, for high-grade gliomas (HGGs), it has been widely accepted that radiologic evaluation is mandatory for distinguishing between tumour residuum/recurrence and therapy-induced changes. These can possess overlapping imaging features including contrast enhancement, surrounding oedema and mass effect, and so discrimination can be very challenging. As a result, there has been increasing focus on accurate assessment of post-treatment imaging.^1,2^ In this paper, we provide a review of the current treatment and imaging strategies for LGG and HGG surveillance, emphasising their shortcomings and challenges and the potential of advanced imaging approaches in clinical evaluation.

### Standards of care and current surveillance imaging approaches

Therapeutic approaches to date for HGGs comprise maximum safe excision followed by radiation and adjuvant temozolomide. However, no standardised scheme has been followed for the management of the inevitable tumour recurrence that leads to almost no improvement in the survival rates of patients with glioblastoma over time.^3^ In the last few years, a novel treatment regimen targeting the immune system of the CNS has been evolving that comprises vaccination therapy and checkpoint inhibitors for Glioblastoma.

For years, MRI has been considered the gold-standard imaging modality for gliomas’ evaluation with several trials conducted to regulate such an approach. In 1990, the Macdonald criteria depended mainly on tumour contrast enhancement using quantitative bidirectional size measurement for brain tumour assessment.^4^ Yet, gadolinium enhancement alone is not reliable for predicting tumour aggressiveness as emerging treatment such as cytotoxic radiotherapy, can cause increased enhancement without disease progression. Additionally, antiangiogenic drugs can result in reduced enhancement independent of any anti-tumour effect.

**Figure 1.**
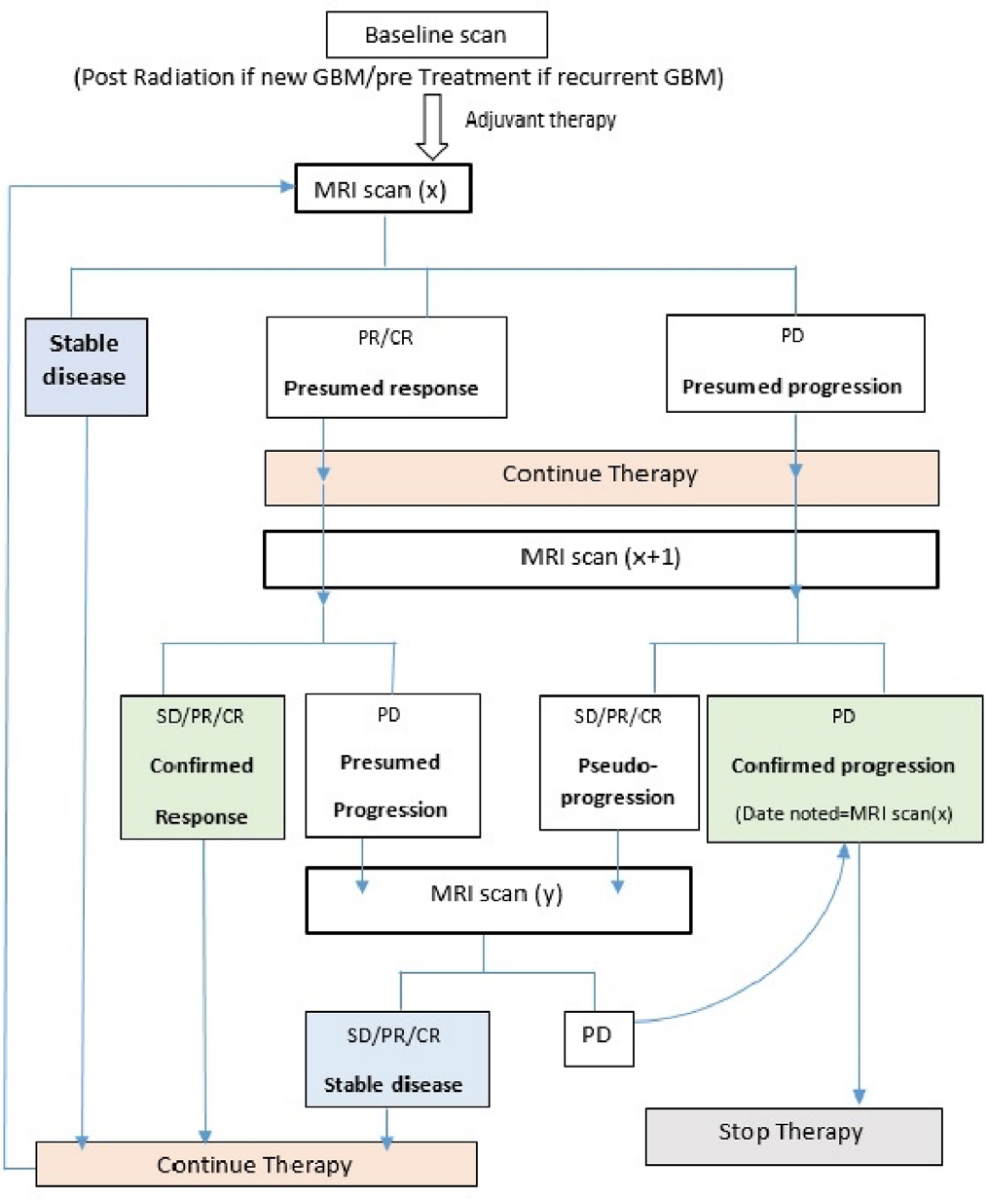
Flow chart for glioma treatment response including pseudoprogression. CR, complete response; PD, progressive disease; PR, partial response; SD, stable disease.

As a result, the Macdonald criteria was updated in 2010 to become the RANO (Response Assessment in Neuro-Oncology) criteria, taking into consideration non-enhancing tumour areas, pseudoresponse and pseudoprogression phenomena along with introducing four main categories of tumour response: progressive disease (PD), partial response (PR), complete response (CR) and stable disease (SD)(Table 1) .^5^ Another limitation of the Macdonald recommendations was that the bidirectional measurement was impractical for evaluating irregular tumours. This prompted the formation of a consensus group which in 2015 provided a list of recommended acquisitions as a part of a standard imaging protocol for gliomas^6^(Table 2). Recently, the RANO criteria was modified by Ellingson and colleagues. They proposed focusing on using volumetric measurements as an alternative way of tumour assessment. Additionally, they introduced a clear definition for non-measurable lesions, to facilitate their exclusion from the decision flow. They have also proposed changing the timeframe for baseline imaging, advocating that this should be defined as the post-radiation scan instead of the post-surgical imaging in newly diagnosed Glioblastoma. In cases of recurrent GBM, they recommended considering the pre-treatment study as the baseline. Finally, a flow chart for glioma response management has been introduced by the mRANO working Group^5^ (Figure 1).

**Table 1.** Definitions for volumetric and bidirectional (SPD) tumour measurements

**Response category**	**PD**	**PR**	**CR**	**SD**
**SPD**	Increase ≥ 25%	Decrease ≥ 50%	Complete disappearance	Decrease < 50% or increase < 25%
**Volumetric variation**	Increase ≥ 40%	Decrease ≥ 65%		Decrease < 65% or increase < 40%

CR, complete response; PD, progressive disease; PR, partial response; SD, stable disease; SPD, sum of products of diameters.

**Table 2.** MRI acquisition techniques recommended for glioma surveillance imaging

	**Pre-contrast *T*_1_WI**	**2D/3D FLAIR**	**2D *T*_2_WI**	**2D DWI**	**Post-contrast *T*_1_WI**
**Scanning sequence**	IR-GRE	TSE/ FSE	TSE/FSE	SS-EPI	IR-GRE
**Plane**	Sagittal/ axial	Axial	Axial	Axial	Sagittal/ axial
**Scanning time**	5–10 min	4–8 min	2–4 min	2–4 min (for 3 b values in three directions)	Same as pre-contrast

2D, two-dimensional; 3D, three-dimensional;DWI, diffusion-weighted image; FLAIR, fluid attenuation inversion recovery; FSE, fast spin echo; IR-GRE, inversion recovery gradient echo; SS-EPI, single shot echoplanar imaging; TSE, turbo spin echo; *T*_1_WI, *T*_1_ weighted image; *T*_2_WI, *T*_2_ weighted image.

Table modified from Ellingson et al.^6^

The various treatment regimes in HGGs are notorious for causing therapy-induced changes including radiation necrosis, pseudoprogression and pseudoresponse. On one hand, radiation necrosis, which usually develops 3–12 months after radiotherapy but could result decades after treatment, represents an aggressive tissue reaction due to blood–brain barrier (BBB) disruption associated with oedema, mass effect and occasionally haemorrhages or calcifications. Principally, radiation necrosis arises at the site of highest radiation dose as an enhancing lesion and can be confused for tumour progression/recurrence in conventional imaging techniques.^7^

On the other hand, pseudoprogression appears as new or increasing areas of enhancement in the 3–4 month-period after radiotherapy and tends to stabilise or partially resolve without any change to the treatment plan.^8^ Hence, It has been acknowledged that pseudoprogression is one of a broad spectrum of radiation-related changes, which range from a subtle radiologic abnormality to late radiation necrosis.^9^

In contrast, with certain anti-angiogenic chemotherapy agents, there can be disappearance of contrast uptake but with recurrence of tumour in the form of a non-enhancing lesion, dubbed “pseudoresponse”.^10^ Both phenomena prompt many uncertainties for caring clinicians and patients and necessitate advanced and/or multiparametric imaging strategies for appropriate interpretation and addressing the best care for patients.

Post-treatment changes, including pseudoprogression, occur more frequently with immunotherapy albeit with a different mechanism than with chemoradiotherapy.^3^ This led to the establishment of a distinct scheme specifically for this novel therapy known as iRANO (immunotherapy response assessment in neuro-oncology) which led to further refinement of glioma response criteria undergoing immunotherapy. Essentially, the scheme considers disease to have progressed only after confirmation with a repeat MRI after 3 months of treatment continuation. A repeat MRI is required in cases of imaging progression with lack of significant clinical deterioration.^11^ Moreover, the iRANO group advised to reduce the use of corticosteroids which may have adverse effects on the potency of immunotherapy.^3^

Response assessment in LGGs has additional limitations. Firstly, many LGGs lack contrast enhancement. This makes the differentiation of tumour tissue from post-treatment changes based only on T2 or FLAIR (fluid attenuation inversion recovery) images more challenging. Secondly, there is a poor correlation between clinical response and post-treatment imaging appearance of LGGs. Another limitation is the progression threshold, which is the minimum percentage increase in the size of a tumour necessary to describe it as progressive. Progression threshold has been defined as reaching >25% in HGGs, whereas LGGs grow very slowly to reach the progression threshold during follow-up. Owing to these limitations, a report from RANO group for assessment of LGGs was established in 2011; that introduced “minor response” category for reductions between 25 and 50% of non-enhancing area in T2 or FLAIR images compared to baseline, in addition to considering the clinical response as a pillar of the treatment (*i.e.* cognitive function, neurologic function and quality of life).^12,13^

Whilst RANO criteria are widely applicable in adult brain tumours’ response evaluation, there are concerns about its suboptimal performance in the paediatric population.^14,15^ The foremost reason behind this is the wide variability in the types of cerebral tumours among children. For instance, there is no distinct subtype representing the majority of the cases, unlike gliomas in the adult’s population, which constitute about 80% of primary brain tumours. Additionally, there is no consensus about the definitions of tumour response to treatment or progression.^14^ In the meantime, there has been a number of clinical trials in the literature investigating and suggesting the most appropriate imaging schemes for paediatric tumours’ response assessment. In 2013, a working group known as RAPNO (response assessment in paediatric neuro-oncology) was founded to establish standardised response assessment for three different paediatric tumours subgroups: HGGs, LGGs and diffuse midline gliomas.^14^ Moreover, two consecutive reviews by D’Arco et al have suggested radiologic assessment protocols for paediatric brain tumours and compared different types of conventional and advanced imaging techniques.^15,16^ However, to date other than the RANO guidelines there have been no settled criteria for assessing paediatric tumours.

Despite the routine use of conventional imaging for assessing glioma response, the non-specific character of the gadolinium enhancement features poses limitations for the differentiation between treatment-induced changes and tumour recurrence. Therefore, advanced physiologic imaging techniques have been widely studied for a better assumption of these different pathologies.

### Overview and critical appraisal of the current advanced imaging modalities

#### MR perfusion

**Figure 2.**
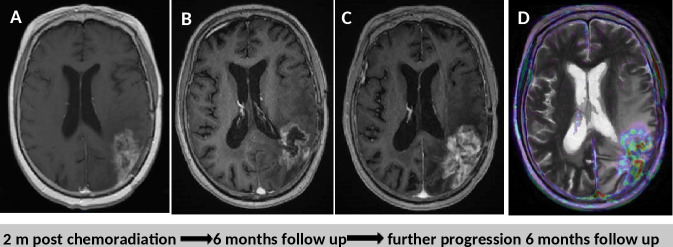
True progression. Treatment surveillance after surgical resection chemoradiation in a patient with GBM. (A, B and C) *T*_1_WI post-gadolinium imaging show progressive increase in the volume of the heterogeneously enhanced residual tumour tissue. (D) Perfusion imaging demonstrates some focally increased cerebral blood volume mostly on the lateral parts of the heterogeneous mass lesion. *T*_1_WI*, T*_1_ weighted imaging.

**Figure 3.**
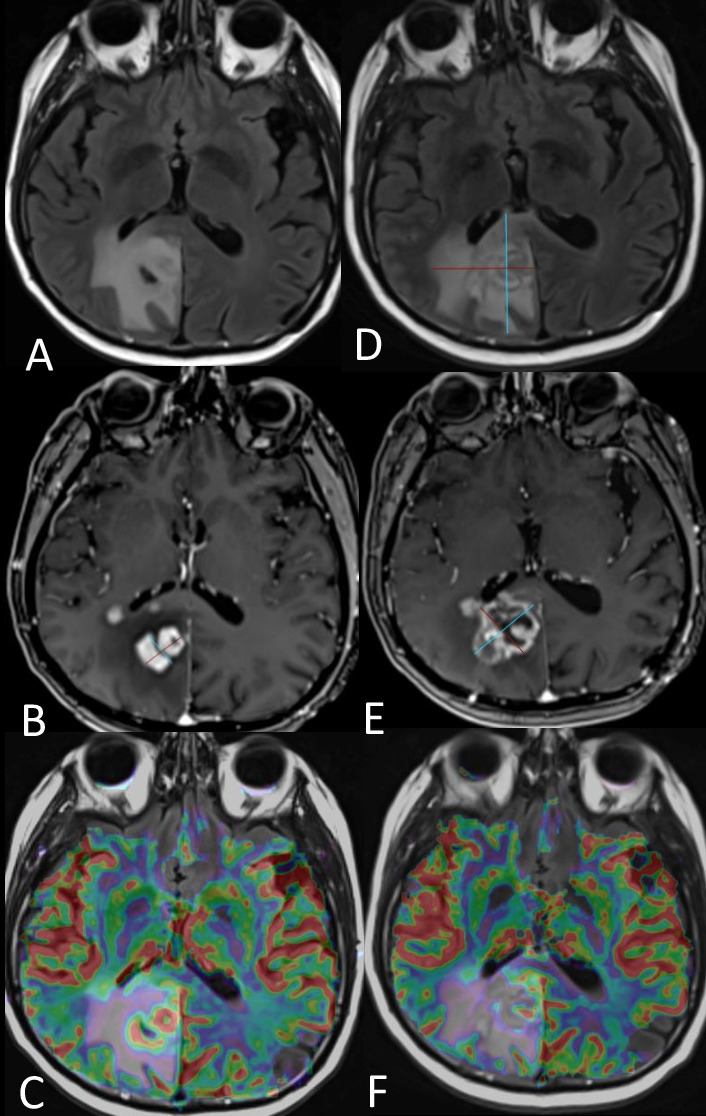
Patient with right occipital glioblastoma underwent diagnostic biopsy and concurrent adjuvant chemoradiation with temozolomide. 6 months after the baseline MRI (A–C), which showed well-circumscribed oedema (arrow in A) with rather solid enhancing (arrow in B) tumour and strong perfusion features in the DSC-derived rCBV map (arrow in C), there is further increase in the oedematous area (arrowhead in D) with expansile partly necrotic tumour mass (arrowhead in E). According to the mRANO criteria, the lesion is classified as preliminary progressive disease but the obvious decrease in the tumour vascularity in the rCBV map (arrowhead in F) demonstrates clearly the pseudoprogression effect. The enhancing lesion was stable in the next MRI follow-up and started resolving 3 months later. DSC, dynamic susceptibility contrast; RANO, Response Assessment in Neuro-Oncology; rCBV, relative cerebral blood volume.

**Figure 4.**
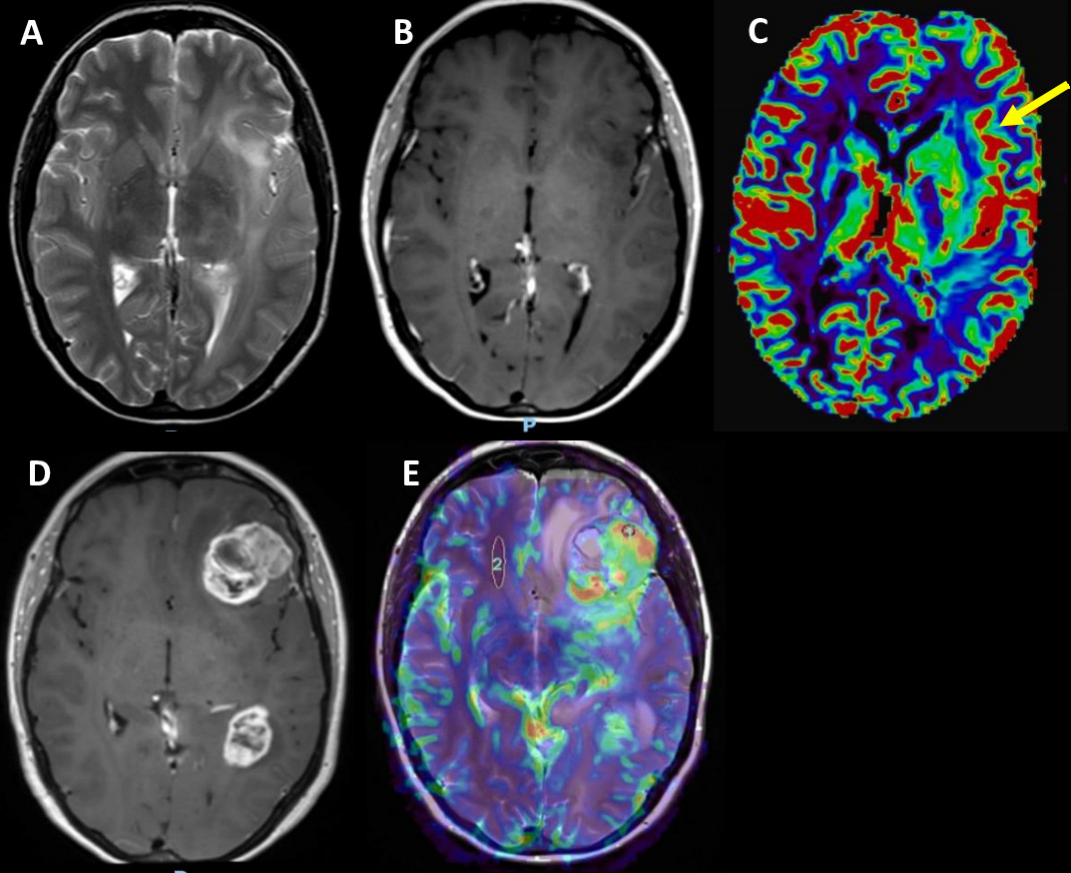
Early progression of a histopathologically verified GBM (IDH wild type). (A) *T*_2_WI displays diffuse abnormal high signal in the left periinsular region. (B) *T*_1_WI with gadolinium shows no significant enhancement within the diffuse glioma. (C) Baseline perfusion image shows foci of high CBV (yellow arrow), eventually suggesting anaplastic foci within diffuse glioma in line with the histopathological diagnosis. (D) *T*_1_WI with gadolinium after 2 months surveillance shows two distinct enhancing lesions. (E) Perfusion image demonstrates remarkably high vascularity in the enhancing lesions with an rCBV ratio of ~9. *T*_1_WI, *T*_1_ weighted imaging; rCBV, relative cerebral blood volume.

**Figure 5.**
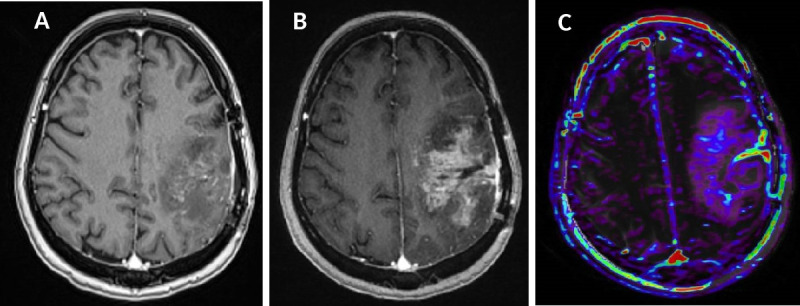
Serial images for a patient with a resected GBM. (A, B) Serial *T*_1_WI post-gadolinium (with a 1-month interval) show increasing irregularly enhanced tissue outlining the resection cavity. (C) Perfusion image demonstrates normal to slightly elevated focal Ktrans values (~0.1) predominantly in line with treatment-related changes (pseudoprogression). *T*_1_WI, *T*_1 _weighted imaging.

**Figure 6.**
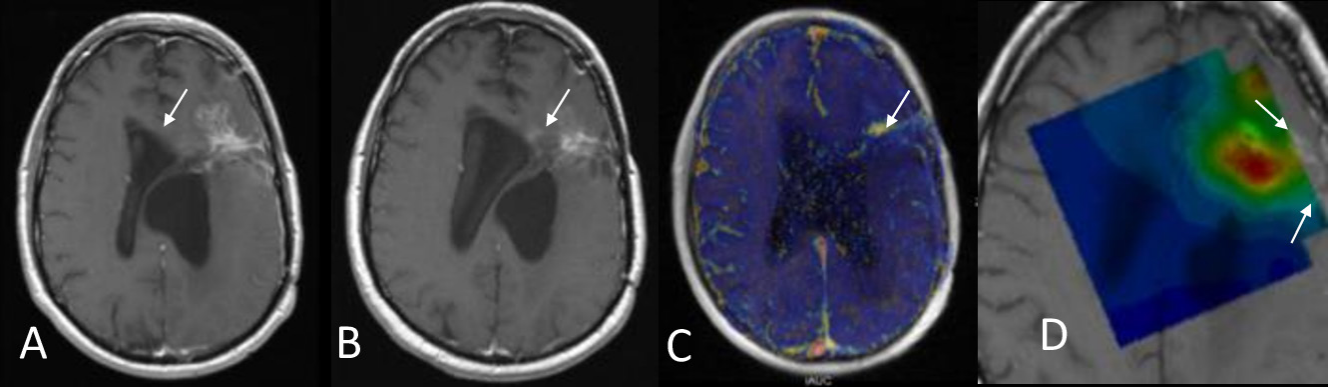
Post-contrast *T*_1_WI (arrow in A) in a patient with recurrent glioblastoma on the left frontal lobe shows the enhancing tumour relapse. The patient underwent combined chemotherapy with temozolomide and bevacizumab showing partial response after 6 months in the post-contrast *T*_1_WI (arrow in B) and in the DCE-derived Ktrans map (arrow in C) due to the anti-neoangiogenic effect of the administered bevacizumab. The MR spectroscopy indicates that the conventional and perfusion MR appearances are largely misleading and show pseudoresponse as the choline map still shows a very high tumour burden (arrows in D). DCE, dynamic contrast enhancement; *T*_1_WI, *T*_1_ weighted imaging.

Since blood perfusion provides oxygen and nutrients to tissues along with being closely tied to tissue function, perfusion disorders have been considered as major sources of medical morbidity and mortality.^17^ MR perfusion, which describes the vascular characteristic of gliomas,includes three main techniques: dynamic susceptibility contrast imaging (DSC), dynamic contrast enhancement (DCE) and arterial spin labelling (ASL). Many studies have focused on DSC, which acquires *T*_2_* weighted images after contrast injection and converts the dynamic signal changes into parametric maps. From these maps, the most common extracted parameter is relative cerebral blood volume (rCBV) (Figures 2–4) . Similarly, volumetric transfer coefficient (*K*^trans^) is considered the most frequently used parameter in DCE^4^ (Figures 5 and 6). Yet, DCE is considered to be understudied owing to its need for more complex pharmacokinetic models that consist of dynamic *T*_1_ imaging before, during and after contrast administration. A systematic review and meta-analysis have probed the diagnostic performance of both DSC and DCE in monitoring gliomas after treatment (Table 3). Including 28 studies, pooled sensitivity and specificity for DSC and DCE indicated that these techniques have good accuracy in discriminating tumour recurrence from treatment induced changes [90% (95% confidence interval, CI: 0.85–0.94) & 88% (95% CI: 0.83–0.92) for DSC and 89% (95% CI: 0.78–0.96) & 85% (95% CI: 0.77–0.91) for DCE, respectively].^24^ Despite its accuracy, DSC – essentially rCBV – could be disrupted with *T*_1_ weighted contrast leakage resulting from BBB destruction. This could result in under- or overestimation of rCBV values inside the tumour,^9^ therefore several clinical trials have been conducted for its correction.^25,26^ Further limitations include the variety of methodologies used, regarding imaging acquisition, post-processing software, analysis methods and the different extracted parameters. This resulted in different thresholds for every metric; *e.g.* rCBV had a threshold range between 0.9 and 2.15 for tumour recurrence detection.^24^

**Table 3.** Summary for MR perfusion studies; sensitivity and specificity for best performing metric

First author and year	Best performing metric	Threshold value	Sensitivity (95% CI)	Specificity (95% CI)
**DSC studies**				
**Kim 20,1424**^18^	90^th^rCBV	>2.37	0.84 (0.66–0.95)	0.95 (0.75–1.00)
**Alexiou 20,1425**^19^	Max rCBV	>2.2	1.00 (0.86–1.00)	1.00 (0.54–1.00)
**Seeger 20,1326**^20^	Mean rCBV	>2.15	0.81 (0.59–0.94)	0.79 (0.53–0.95)
**DCE studies**				
**Bisdas 20,1127**^21^	Median Ktrans	>0.19	1.00 (0.74–1.00)	0.83 (0.36–1.00)
**Chung 20,1328**^22^	Mean AUCR	>0.23	0.94 (0.79–0.99)	0.88 (0.69–0.97)
**Yun 20,1529**^23^	5^th^Ve	>0.18	0.76 (0.50–0.93)	0.88 (0.62–0.99)

DSC: dynamic susceptibility contrast imaging, rCBV: relative cerebral blood volume, Max: maximum, DCE: dynamic contrast enhancement, Ktrans: volumetric transfer coefficient, AUCR: area under curve ratio, Ve: fractional volume of extracellular extravascular space.

ASL is recognised as a non-invasive technique of measuring cerebral blood flow (CBF) using labelled endogenous blood and creates a normalised CBF map as the main parameter to consider.^17^ ASL could be ideal for the long-term follow-up of gliomas following radiation, including those with renal dysfunction.^27^ Jing Ye et al have elaborated the use of ASL in differentiating recurrent gliomas from post-treatment changes. It was found that the normalised CBF ratio (4.45 ± 2.72) was higher in cases of glioma recurrence than that in cases of radiation injury (1.22 ± 0.61) (*p* < 0.01). Moreover, a close linear correlation was found between the ASL and DSC MRI techniques (linear regression coefficient, *R* = 0.85; *p* = 0.005) in the differentiation of recurrent glioma from radiation injury.^28^

### Diffusion-weighted imaging (DWI)

**Figure 7.**
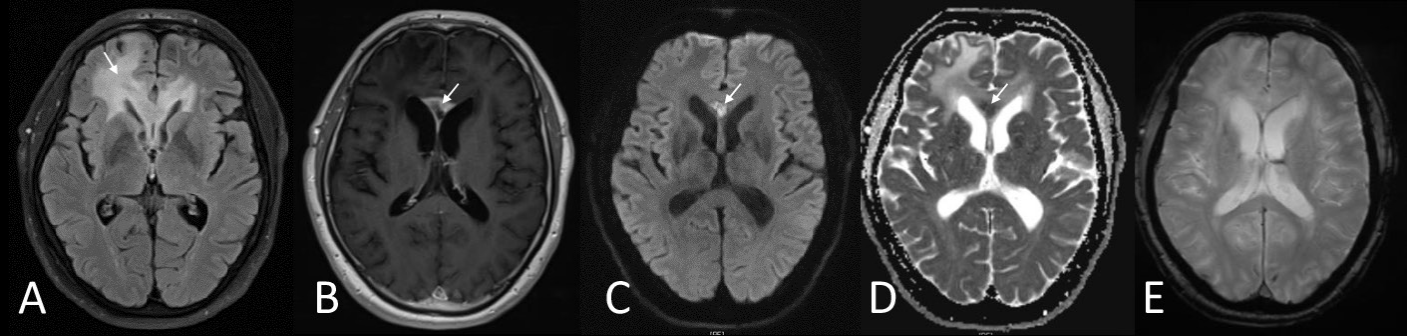
Patient with glioblastoma undertreatment shows extensive vasogenic oedema on the right frontal lobe (arrow in A) with fairly well-circumscribed enhancement on the genu of the corpus callosum (arrow in B). The enhancing region demonstrates markedly restricted diffusion (arrows in C, D) without haemorrhagic features (E) suggesting hence hypercellular tumour recurrence.

**Figure 8.**
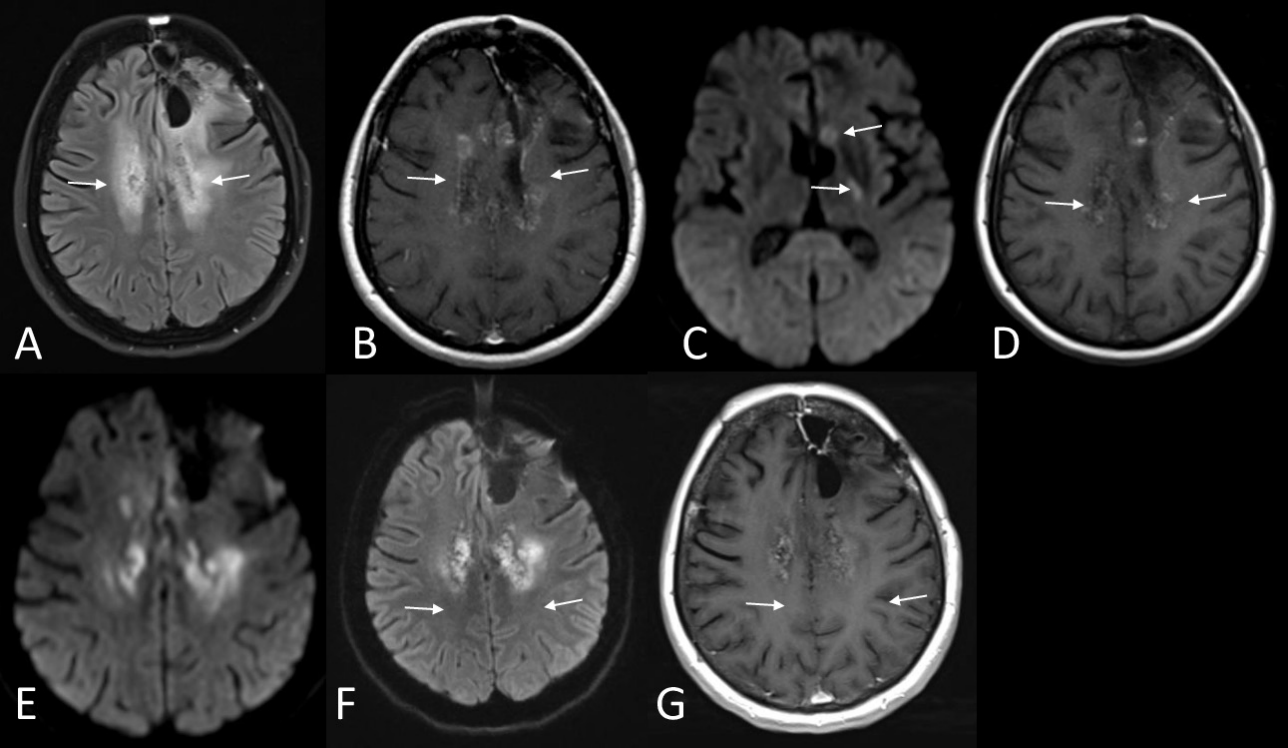
Patient with recurrent glioblastoma, treated with bevacizumab as mono-therapy, shows diffuse oedema in the *T*_2_ FLAIR images (arrows in A) surrounding the surgical cavity. The post-contrast *T*_1_WI show patchy, confluent enhancement in the area with abnormal *T*_2_ signal intensity (arrows in B) and a few areas with restricted diffusion (arrows in C). 6 months later, the gadolinium enhancement has decreased (arrows in D) suggesting hence response. The diffusion restriction (arrows in E) appears to be increasing in the areas of the previous patchy enhancement and the DWI retains its high signal 6 months later (arrows in F) confirming partial response to therapy. The contrast enhancement has also stable appearances (G). The diffusion restriction is believed to represent ischaemic tissue with coagulative necrosis. DWI, diffusion-weighted imaging; FLAIR, fluid attenuation inversion recovery; *T*_1_WI, *T*_1_ weighted imaging..

Apparent diffusion coefficient (ADC) is derived from DWI as a quantitative metric for water molecules’ diffusivity within the tumour^4^ and has been widely used for glioma assessment, grading and treatment monitoring (Figures 7 and 8). Wong et al concluded that mean ADC showed the best performance in differentiating true from pseudoprogression (cut-off value 1200 × 10⁻⁶ mm²/s at sensitivity 80% and specificity 83.3%).^29^ Another study by Bulik et al showed perfect (100%) sensitivity and specificity at 1300 × 10⁻⁶ mm²/s cut-off value.^18^ Meanwhile, Chu et al probed two different b values for DWI and stated that the fifth percentile ADC acquired from the higher b value (3000 s/mm²) displayed the best diagnostic accuracy (sensitivity 93.3% , specificity 100% at cut-off value 645 × 10^−6^ mm²/s).^19^ A meta-analysis explored seven studies that investigated the feasibility of DWI in differentiating true from pseudoprogression.^20^ They concluded that DWI has conveyed better accuracy than conventional imaging in discriminating true from pseudoprogression (pooled sensitivity and specificity = 71 and 87% respectively)(Table 4). However, the same meta-analysis has shown that the diagnostic performance of DWI is subordinate compared to MR perfusion and magnetic resonance spectroscopy (MRS).^20^

**Table 4.** Summary for DWI quantitative assessment studies; cut-off values, sensitivity and specificity for best performing value

First author and year	Best performing metric for differentiating true progression from pseudoprogression	Cut-off value10⁻⁶ mm²/sec	Sensitivity	Specificity
**Bulik et al, 2015^18^**	Mean ADC value	1300	100%	100%
**Lotumolo et al, 2015^19^**	Mean ADC value	1000	81.8%	69.2%
**Chu et al, 2013^20^**	Fifth percentile ADC1000 value	929	73.3%	73.3%
	Fifth percentile ADC3000 value	645	93.33%	100%
**Lee et al, 2012^30^**	Mean ADC value	1200	80%	83.3%

ADC, apparent diffusion coefficient; DWI, diffusion-weighted imaging.

In addition to variability in MRI hardware and acquisition techniques, the heterogeneous ADC values seen in the same tumour tissue, due in part to viable tumour cells neighbouring tissue necrosis, may result in a non-representative value for the mean ADC within a glioma.^9^ Addressing these challenges, Ellingson et al investigated functional diffusion maps (fDM) which assumed tumour non-homogeneity and calculated voxel wise changes in ADC. In the lights of predicting gliomas’ response, they suggested that tumours with more tissue volume of decreased ADC (>20% of T2FLAIR or >15% of contrast uptake areas) within 4 weeks from completing chemoradiotherapy, tend more to have worse prognosis compared to lesions comprising less tissue volume with decreased ADC or more tissue volume with increased ADC.^21,22^ Moreover, several trials have studied the application of histogram analyses for ADC maps with different diffusion gradient b values up to 3000 mm²/s, and was found to be promising using high b values and histogram analysis of ADC values.^19^

Recently, other emerging diffusion-based techniques,including intravoxel incoherent motion (IVIM) and diffusion kurtosis imaging (DKI) are being investigated for their potential inglioma grading.^23,30,31^ IVIM is a technique that acquires quantitative perfusion data by means of DWI using several b-values.^32^ Miyoshi et al investigated the role of IVIM in identifying recurrent tumour after chemotherapy. They showed that relative diffusion coefficient (rD), an IVIM extracted parameter, had a good correlation with contrast enhanced T1 and DWI. Low rD values indicated tumour progression and the authors concluded that this was more useful than ADC and ASL values.^32^ DKI is a simple continuation to diffusion tensor imaging (DTI) that utilises higher b-values in order to assess the non-Gaussianity within tissue microenvironment.^33^ Several studies have been investigating the feasibility of DKI in glioma grading; however, there is paucity of trials concerning its role in post-treatment evaluation. A single clinical trial recently compared its role in monitoring gliomas response against the role of monoexponential diffusion (ADC). They concluded that complex diffusion models as DKI and stretched exponential diffusion did not add any significant data in predicting overall survival, compared to conventional DWI.^34^ Albeit, further scientific trials with larger cohorts in both IVIM and DKI are warranted for better assessment of their actual value in gliomas’ surveillance.

Furthermore, DTI has been increasingly used in glioblastoma characterisation utilising anisotropic measures as fractional anisotropy (FA), planar anisotropy (CP), spherical anisotropy (CS) and linear anisotropy (CL) with only few studies explored its value in assessment of gliomas’ response to treatment.^35,36^ Wang et al studied DTI ability to differentiate true from pseudoprogression, where their results comprised that planar anisotropy was the single best predictor of true progression (AUC: 0.84, 95% CI: 0.72–0.96).^35^ While AUC value has soared up to 0.905 (95% CI: 0.81–1.00) when three parameters (FA, CP and maximum cerebral blood volume CBV) were combined together.^35^

### Magnetic resonance spectroscopy (MRS)

**Figure 9.**
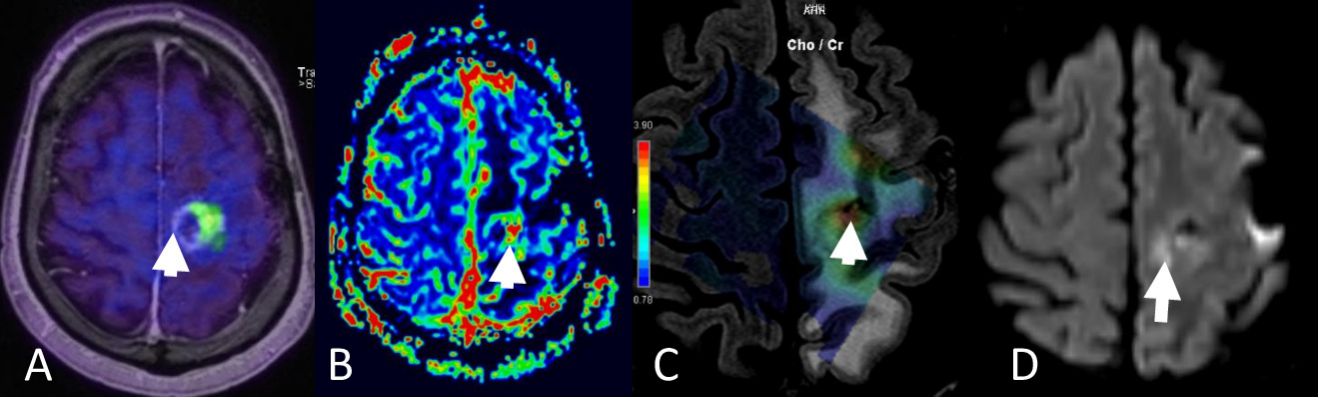
Patient with left precentral anaplastic (WHO Grade 3) glioma treated with partial resection and adjuvant concurrent chemoradiation. Three months after treatment end, the patient started experiencing progressing hyperintense *T*_2_WI signal changes in the left frontoparietal hemisphere and undergoes multiparametric hybrid MR-PET imaging with methionine. The enhancing lesion shows a tracer ”hot-spot” (arrow in A) with increased vascularity (arrow in B, DSC-derived relative cerebral blood volume map) and increased focal cell proliferation rate denoted by the increased ratio of choline to creatine in the MR spectroscopy (arrow in C). Interestingly, the diffusion weighted imaging shows pathological signal (arrow in D) on the medial part of the lesion, which appears “cold” on the other biomarkers’ maps. The finding probably reflects the different stages of tumour metabolism and growth captured by the different imaging modalities in a complementary way. DSC, dynamic susceptibility contrast; MR-PET, magnetic resonance-positron emmision imaging; *T*_2_WI *T*_2_ weighted imaging.

MRS examines the spatial dispersion and different metabolites’ concentration in tumour tissue and has been used in brain tumours’ assessment^18^ (Figure 9). A meta-analysis by Zhang et al included 18 studies to probe the role of MRS in differentiating recurrent glioma from radiation necrosis. They have found that the Choline/N-Acetyl Acetate (Cho/NAA) and Choline/Creatine(Cho/Cr) ratios, showed moderate diagnostic accuracy; pooled sensitivity and specificity for Cho/NAA 88 and 86%. However, they have recommended that decision-making should not be dependent solely on MRS.^37^ Another meta-analysis compared the performance of MRS with MR perfusion and DWI in discriminating true from pseudoprogression by analysing nine studies, which concluded that MRS displayed the highest diagnostic performance (pooled sensitivity and specificity 91 and 95%).^20^

Comparable to DWI, heterogeneity inside the tumour environment has contributed to the relatively limited application of MRS, particularly when using single voxel techniques. Therefore, multivoxel techniques have been proposed, with initial studies demonstrating better diagnostic accuracies (83.3 and 96.2%) in differentiating tumour tissue from necrosis compared to single voxel techniques.^38,39^ In one study, cut-off value for Cho/NAA in discriminating true from pseudoprogression was ≥1.4 when sensitivity and specificity were 100 and 91.7%.^18^ Nevertheless, there remain plenty of constraints, including the lack of standardised imaging acquisition, software processing and data analysis with fluctuating cut-off values.

In the meantime, a recently probed MRS-onco metabolite has been unfolded, known as 2-hydroxy glutarate (2HG). 2HG accumulation results from isocitrate dehydrogenase (IDH) genetic mutation in LGGs and this, combined with the integration of IDH mutation status in the WHO 2016 gliomas’ reclassification, suggests that the metabolite could be used as a state-of-the-art biomarker for IDH-mutant gliomas.^40^ Additionally, it has been established through several clinical trials that 2HG can be identified by MRS.^41,42^ Two studies have conveyed the significance of integrating 2HG-MRS into monitoring IDH-mutant gliomas. Both groups concluded that mean 2HG values significantly decrease in the post-treatment scans and recommended the technique to be incorporated in the daily practice for assessing IDH-mutant gliomas.^40,42^

### Chemical exchange saturation transfer (CEST)

**Figure 10.**
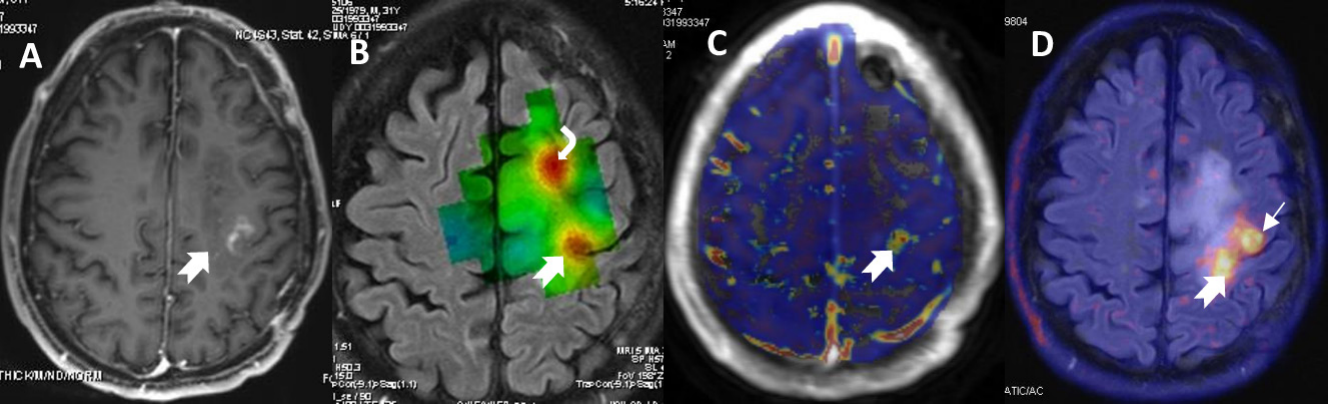
Patchy enhancement (notched arrow in A) in the left frontal precentral area in a patient with anaplastic oligodendroglioma 6 months after treatment end. The choline metabolic map (B) from MR spectroscopy (Chemical Shift Imaging) shows significantly high amount of this metabolite (notched arrow and curved arrow) in the tumour area indicating possible recurrence. The DCE-Ktrans map (C) shows that the precentral area has increased permeability values (notched arrow) and the hybrid MR-PET imaging with methionine (notched arrow in the fused PET with T2FLAIR images in D) corroborates the suspected tumour recurrence. Further avid tracer uptake anterior to the enhancing lesion (arrow in C) denotes tumour relapse without disruption of the blood-brain-barrier or increased neo-angiogenesis. DCE, dynamic contrast enhancement; FLAIR, fluid-attenuated inversion recovery; MR-PET, magnetic resonance-positron emission tomography.

CEST is a relatively new MRI technique that has the potential to image chemical compounds present in tissue at concentrations that are too low to affect the contrast resolution of conventional imaging or to be directly detected with MRS at typical water imaging resolution.^43^ A common characteristic of many *in-vivo* chemical metabolites, involved in normal physiological and pathological processes, is that their hydrogen nuclei resonate at different frequencies to those of protons in water molecules. This may be detected by MRS, but is often limited in clinical practice by their low concentrations *in-vivo* and limited sensitivity at the clinical MRI scanner field strengths (typically ≤3 T).^43^ Because of this, there has been a significant interest in CEST imaging. The signal in CEST is generated by chemical exchange of ^1^H protons of the solute of interest with those of water. As a result, the magnetisation is transferred to the large water proton pool over time leading to a decrease in water signal, which can provide an indirect measure of the concentration of the solute(s) of interest. It can therefore be used to measure the concentration of *in-vivo* metabolites that can act as biomarkers for tumour evaluation and monitoring^44,45^ (Figure 10). These endogenous CEST agents include amide group of proteins and peptides known as amide protein transfer (APT), amine group of small metabolites (such as glutamate and creatine) and finally hydroxyl group on glycogen and glucose.^44^ Referring to the high cellular content of aggressive gliomas, a higher concentration of proteins and peptides are expected to be detected within the region of the tumour.^45^ Zhou et al found a significantly higher APT signal in gliomas compared to normal brain tissue in a rat model which declined significantly after radiotherapy (*p* < 0.001).^45^ Okuchi and colleagues conducted a systematic review on the use of endogenous CEST for the diagnosis and therapy response assessment of brain tumours.^46^ They found four studies that sought to assess the potential of CEST to differentiate tumour recurrence from treatment related changes, including a total of 161 glioma patients imaged on 3 T MRI systems.^47–50^ All studies used APT imaging. APT signals were found to be significantly higher in regions demonstrating tumour progression compared to areas of therapy-induced changes, with reported high diagnostic accuracies (AUC 0.88–0.98). Park reported greater diagnostic accuracy for APT compared to PET imaging using 11C methionine (11C-MET).^48^

Two further studies have investigated the potential of CEST in assessing gliomas’ response and predicting their prognosis.^46,51,52^ Regnery et al reported that pre-treatment tumour signal in Nuclear Overhauser Enhancement - Lorentzian difference differed significantly based on responsiveness to first-line treatment, with an AUC of 0.98.^51^ Harris et al showed that tumours that were acidic at baseline, as defined by *a* > 50% region of positive CEST asymmetry at 3.0 ppm, had a significantly longer progression free survival (PFS) compared to patients whose tumours weren't acidic (log-rank, *p* < .0001; median PFS for acidic tumours *vs* non-acidic tumours = 125 days *vs* 450 days).^52^ This suggests that APT CEST may be able to reflect baseline and dynamic changes in lesion acidity as an imaging biomarker of residual/viable glioblastoma.

Despite being a promising technique, CEST has a number of challenging technical considerations including the vendor field strength, field homogeneity, concentration of metabolites and finally RF pulse sequences^44^ before drawing any clinically pertinent conclusions from the data.

### Nuclear medicine imaging

A valuable complementary molecular technique to MRI in the clinical management of gliomas is PET. PET has been widely incorporated into neuro-oncology assessment, particularly with post-immunotherapy assessment when MRI findings are often equivocal. ^18^F-flu-deoxyglucose is used to be the most widely utilised tracer. Moreover,FDG-PET tends to be useful in differentiating radiation necrosis from tumour recurrence.^53^ A meta-analysis of 16 studies on FDG-PET showed a good accuracy in differentiating glioma recurrence from treatment-induced changes (pooled sensitivity and specificity 0.77 and 0.78 respectively).^53^ That said, variable ranges of sensitivities and specificities have been established in the literature,^54^ which may be due to the reduced contrast between viable tumour and normal brain tissue uptake of FDG.

11C-choline (CHO), another radiotracer, has been proposed to be helpful in brain tumour imaging. Takenaka et al performed a comparative study between CHO-PET and FDG-PET performance in differentiating glioma recurrence from radiation necrosis and reported a sensitivity/specificity of 73.5%/97.5% and 76.5%/75% respectively.^55^

**Figure 11.**
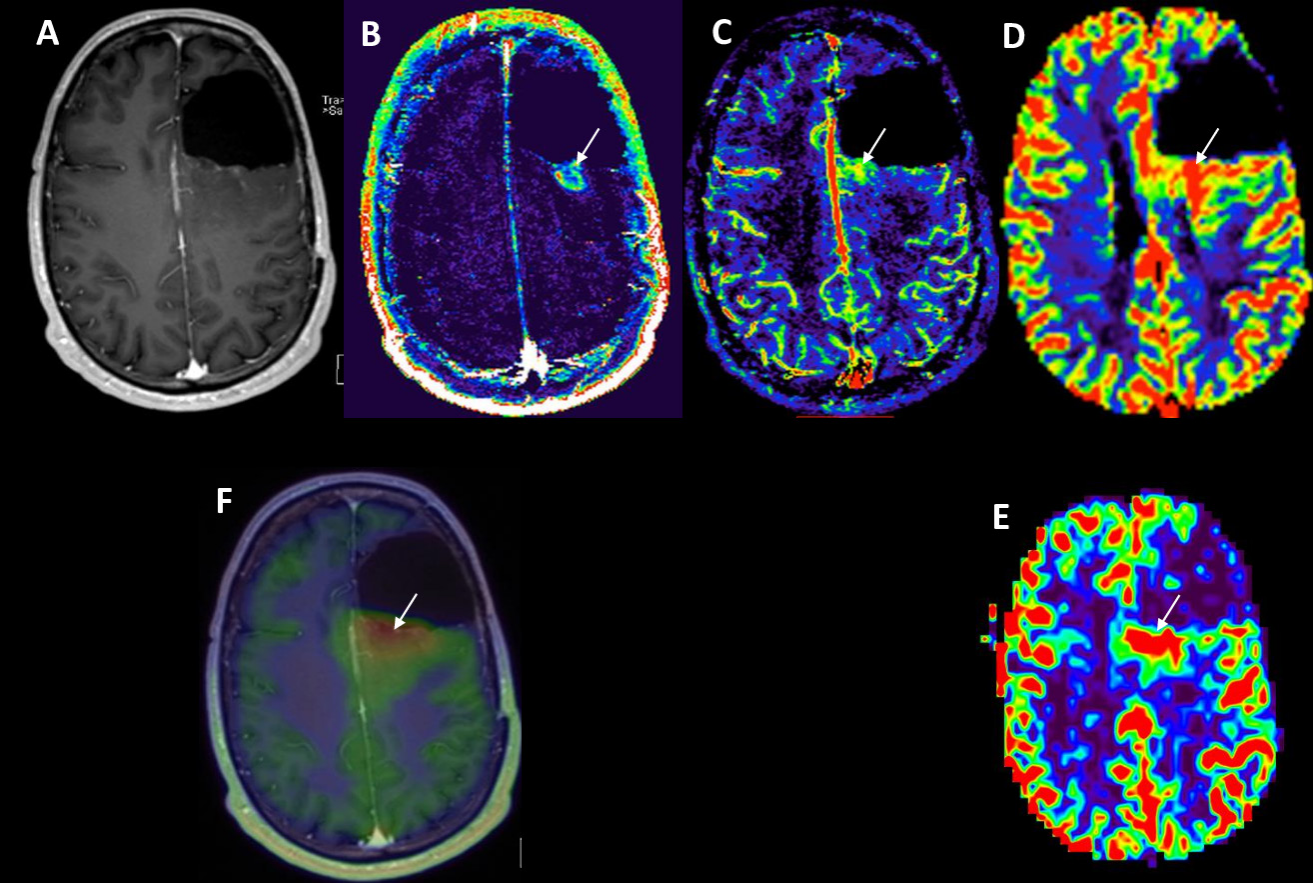
Multiparametric hybrid MR-PET imaging in a patient with IDH-mutant, WHO Grade 3 astrocytoma on the left frontal lobe treated with surgery and adjuvant chemoradiation. Progressive signal changes in the T2FLAIR images (not shown) with some tiny patchy enhancement (A) prompted the comprehensive MR-PET exam. Pathological changes related to neovascularity are visible in the permeability map (B) and intravascular blood volume (C) derived from the DCE with slight incongruence in their spatial distribution zone, reflecting the different pathophysiology that these biomarkers may capture. The intravascular blood volume map is more in line with the DSC-derived rCBV map (D) and the blood flow map from the arterial spin labelling measurement (E). (F) The fused post-contrast *T*_1_WI with the FET-PET image shows avid tracer uptake in the area of hypervascularity. FLAIR, fluid-attenuated inversion recovery; PET, positron emission tomography; *T*_1_WI *T*_1_ weighted imaging.

More specific tracers, namely amino-acid tracers, characterised by their lower uptake in normal brain tissue than FDG,^56^ have also been probed in gliomas’ management. 3, 4-dihydroxy-6-F-flu-l-phenylalanine (FDOPA), 11C-methyl-L-methionine (MET) and 18F-fluethyl-L-tyrosine (FET) are the most frequently used non-FDG amino-acid agents^57^ (Figure 11). Takenaka and colleagues compared between the diagnostic accuracy of MET-PET and PET using other radiotracers as CHO and FDG in identifying post-treatment features in gliomas, the study resulted in an area under the curve (AUC) of MET-PET higher than that of CHO-PET and FDG-PET (AUC using MET, CHO and FDG 0.92, 0.81 and 0.77 respectively).^55^ Eventually, they concluded that MET-PET is more useful in identifying glioma recurrence than FDG-PET and CHO-PET.^55^

A single study investigated the diagnostic accuracy of FET-PET in defining glioma recurrence and reported 93% sensitivity, 100% specificity and 93% accuracy. When selecting a tracer, consideration also needs to be given to the radionuclide’s half-life. In this respect ^18^F-FET, includes ^18^F with a half-life of 109 min, has a logistical advantage over 11C-MET, that due to the 20 min half-life of the 11C, is limited to use in PET centres with an onsite cyclotron unit.^56^ Additionally, FET has more affinity for tumour cells, and has shown promise in gliomas’ surveillance.^56^ Another meta-analysis compared the performance of PET using FDG and non-FDG amino-acid tracers essentially 11C-MET; as data from the remainder non-FDG amino acids as FET and ^18^F-fluthymidine (FLT) was insufficient to perform an analysis.^53^ They concluded that both FDG and MET-PET have shown moderately good accuracies in differentiating glioma recurrence from treatment-induced changes (pooled sensitivity and specificity for FDG-PET are 0.77 and 0.78 and for MET-PET are 0.70 and 0.93 respectively).^53^ Li and colleagues compared between the diagnostic accuracy of FLT and FDG-PET. They found the pooled sensitivity, specificity and overall diagnostic accuracy using FDG to be 0.78 (95% CI 0.69–0.85), 0.77 (95% CI 0.66–0.85) and 0.84 (95% CI 0.81–0.87) respectively, while with FLT to be 0.82 (95% CI 0.51–0.95), 0.76 (95% CI 0.50–0.91) and 0.85 (95% CI 0.81–0.88) respectively.^58^ They therefore suggested that FLT-PET has a moderately better diagnostic performance than FDG-PET in distinguishing glioma recurrence from post-treatment changes.^58^ Meanwhile, FLT that relies on determining cellular proliferation due to its role in DNA synthesis pathway rather than detecting the metabolic activity as in FDG, is normally available in little portions within the brain parenchyma unless the BBB is disrupted as in cases of HGGs.^59^ This means that the feasibility of utilising FLT-PET in LGG surveillance would not be reliable owing for its failure to cross-intact BBB in case of LGG.

The most recently published guidelines by the RANO group, in conjunction with the Society of Nuclear Medicine and Molecular Imaging (SNMMI), the European Association of Nuclear Medicine (EANM) and the European Association of Neuro-oncology (EANO), have comprised the clinical practice of PET in glioma imaging including tumour’s recurrence.^54^ They illustrated several informative details about the performance and interpretation of PET imaging (including for instance well-known pitfalls for each tracer and different tracers’ thresholds). These guidelines will hopefully aid in the inclusion of molecular brain imaging into the routine clinical assessment of gliomas post-treatment, particularly when advanced MRI findings are inconclusive.^54^Table 5 lists the diagnostic accuracy for most of the advanced imaging techniques available in the literature.

**Table 5.** Summary for advanced imaging techniques

Advanced imaging techniques	Sensitivity %	Specificity %	Accuracy/AUC	Best performing parameter	Cut-off value
**DSC^20^**	81.0	76.9	79.4 (accuracy)	rCBV	2.15
**DCE^21^**	100	83	0.976 (AUC)	Median *K*^trans^	0.19 *
**DWI**^65^	73.7	70	0.779 (AUC)	ADC	<0.00149 **
**MRS^20^**	70	78.6	73.5 (accuracy)	Cho/Cr	1.07
**FDG-PET** ^66^	100	75	0.98 (AUC)	FDG ratio lesion: white matter	1.82
**FET-PET** ^67^	74	91	75 (accuracy), 0.91 (AUC)	TBRmean	>2.0
**FDG-PET/MRI**^68^	n/a	n/a	0.935 (AUC)	ADCmean, Cho/Cr, TBRmean	n/a

unit = mm²/s *unit = s-¹, **ADC, apparent diffusion coefficient; AUC, area under the curve; DCE, dynamic contrast enhancement; DSC, dynamic susceptibility contrast; DWI, diffusion-weighted imaging; FDG, fludeoxyglucose; MRS, magentic resonance spectroscopy; PET, positron emission tomography ; TBR:tumour/brain ratio;rCBV, relative cerebral blood volume.

### Multiparametric approaches

**Figure 12.**
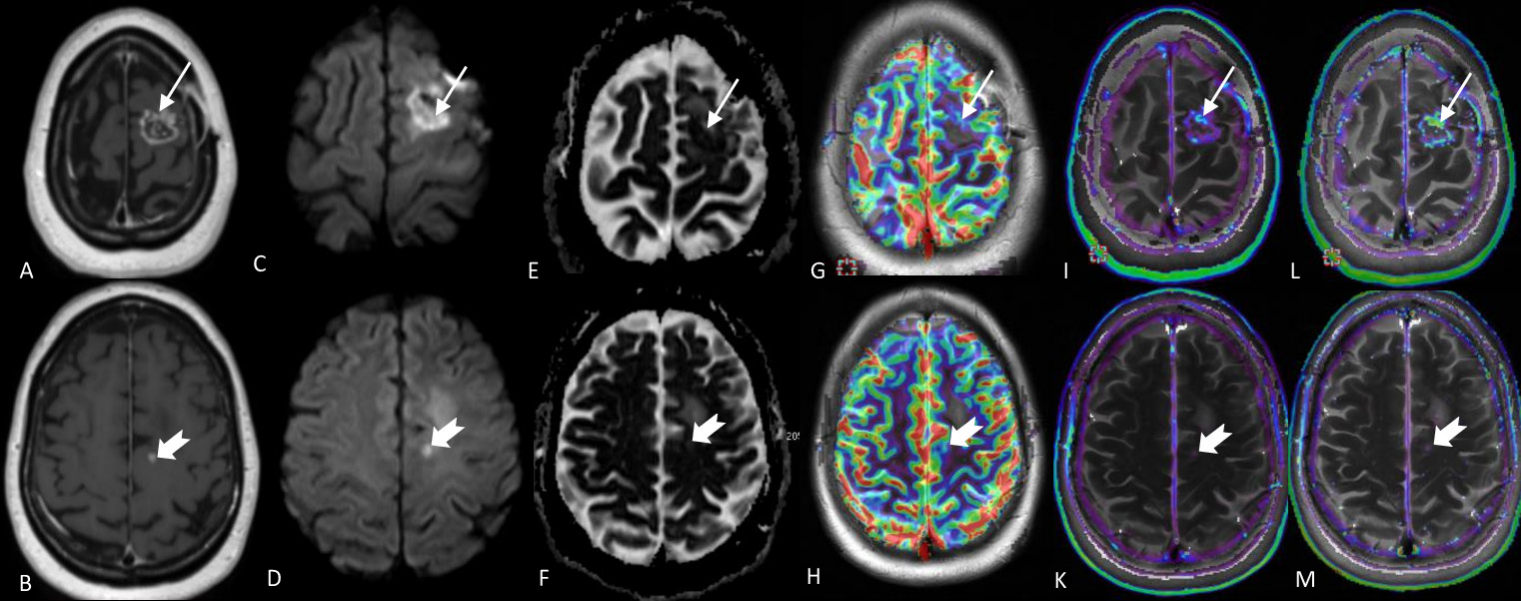
Multiparametric MRI in a patient with GBM on the left frontal lobe treated with surgery and adjuvant radiation with concurrent temozolomide. The arrows on the images in the upper row demonstrate the ring-enhancing treated tumour bed (A) with strong diffusion attenuation (C, E) due to presumable necrotic material and blood and punctate increase in the relative cerebral blood volume (G) and permeability-Ktrans (J) maps. The interstitial volume map (L) shows significant, treatment-related increase in the extravascular extracellular space (ve). The adjacent slice (B) shows punctate enhancement (notched arrow) with strong diffusion attenuation and perifocal oedema (D,F). The enhancing focus shows also increased vascularity in the relative cerebral blood volume (H) and permeability- Ktrans (K) maps, though its conspicuity is less on the DSC imaging (notched arrows). The lesion demonstrates also pathological “ve” values (M). The final diagnosis suggests predominantly treatment-related changes with foci of tumour residual/recurrent disease. DSC, dynamic susceptibility contrast.

Owing to the variability in sensitivity and specificity in the various advanced imaging methods described above, there has been a lot of interest in combining the data from one or more different modalities in the assessment of glioma treatment response (Figure 12). Two studies investigated the ability of MRS, DWI and perfusion imaging separately and combined together to differentiate between glioma recurrence and treatment-induced changes. One group reported an improved diagnostic performance for the multiparametric approach compared to standalone techniques; in which 96.6% for combined MRS, DWI and perfusion *vs* 79% for MRS alone, 89% and 86% when considering combined MRS and perfusion or MRS and DWI respectively. Similarly the other study concluded better diagnostic accuracy of 93% for combined approaches *vs* 86.7%, 86.7%, and 84.6% for separately used DWI, perfusion and MRS respectively.^1,60^ Imani et al used a hybrid MRI-PET technique and probed the use of MRS with FDG-PET in glioma monitoring, finding a diagnostic accuracy of 83%.^61^

### Summary

In summary, post-therapeutic follow-up imaging of gliomas has been considered crucial in response assessment and differentiating tumour recurrence from therapy-induced changes that have been attributed to the various treatment strategies described above. The development of RANO and its modified version had its impact in categorising the different treatment strategies responses that are of lower diagnostic efficiencies in children where RAPNO is used.

Reviewing the studies, that have emphasised the role of current advanced MRI techniques and nuclear imaging ones in glioma surveillance, has revealed the variable accuracies, sensitivities and specificities resulted from studying either each advanced technique or when compared to each other with improvement in diagnostic performance when using multiple modalities together. However, there remain many challenges facing such advanced techniques before they can be incorporated into the routine clinical practice. Further studies are therefore indicated, particularly with larger and more homogeneous as well as more diverse data sets to improve these methods’ diagnostic efficacy.
